# Association between cervical MRI findings and patient-reported severity of headache in patients with persistent neck pain: a cross-sectional study

**DOI:** 10.1186/s12998-025-00600-4

**Published:** 2025-09-01

**Authors:** Dorthe S. Ziegler, Maria Emilie Iversen, Kasper S. Hvid, Kristina B. Dissing, Rikke K. Jensen

**Affiliations:** 1 Medical Spinal Research Unit, University Hospital of Southern Denmark, Sygehusvej 24, 6000 Kolding, Denmark; 2 Department of Regional Health Research, University of Southern Denmark, Odense, Denmark; 3 Faculty of Health Sciences, University of Southern Denmark, Odense, Denmark; 4 Chiropractic Knowledge Hub, Odense, Denmark; 5 Department of Sports Science and Clinical Biomechanics, University of Southern Denmark, Odense, Denmark

**Keywords:** Headache, Cervical spine, MRI, Degeneration, Aggregate score, Neck pain

## Abstract

**Background:**

Neck pain and headaches often co-occur, and the presence of degenerative cervical Magnetic Resonance Imaging (MRI) findings has been associated with the presence of headaches. However, previous studies have not provided conclusive evidence about their association, and imaging studies examining the associations between headache severity and MRI findings have been suggested. This study aims to investigate the associations between independent variables, single MRI findings, and an aggregate score of MRI findings, and the outcome variable, headache severity.

**Methods:**

This cross-sectional study examined patients with neck pain and headaches in specialist care. MRI findings and outcome measures were collected at the time of clinical entrance between 2011 and 2014. The headache severity was assessed using the Neck Disability Index questionnaire. Ten degenerative MRI findings were routinely evaluated, and an overall score was derived by aggregating single findings across levels C2–C7. Univariate and multivariable ordinal logistic regression analyses assessed the associations expressed as odds ratios (OR) and 95% confidence interval (95% CI).

**Results:**

A total of 574 patients were included. Higher headache severity was significantly associated with female sex and younger age. The presence of single cervical MRI findings was linked to lower odds of severe headaches (ORs < 1), and having two or three findings further decreased the likelihood (OR 0.40, 95% CI 0.23–0.68) compared to having none. A sensitivity analysis assessed the OR estimates for the aggregate score as robust.

**Conclusions:**

This study showed that, among patients with persistent neck pain referred to secondary care, degenerative MRI findings in the cervical spine were inversely associated with headache severity. The association between an aggregated score of MRI findings and headache severity was stronger than that of single findings. These findings reflect associations observed within a selected clinical population and warrant further investigation in populations with differing symptom profiles.

**Supplementary Information:**

The online version contains supplementary material available at 10.1186/s12998-025-00600-4.

## Background

Most people have experienced headaches, with a lifetime prevalence of up to 66% or more in the general population and a point prevalence of up to 37% [1–3]. This makes it one of the top five causes of global disability-adjusted life years [4], with a significant socioeconomic impact [3].

Some headaches seem to originate from musculoskeletal disorders in the cervical spine [5], although the pathophysiological mechanisms are not yet fully understood. Neck pain and headaches often co-occur [6–9], and the presence of degenerative or abnormal findings using Magnetic Resonance Imaging (MRI) in the cervical region, such as disc herniation, muscle atrophy or deviations in the spine alignment, has been variously associated with the presence of headaches [10–13]. Also, headache intensity has been found to vary according to the level of neck disability [14], and fewer and less intense headaches have been reported after injections or surgical treatment targeting the cervical spine, supporting the notion that the aetiology of some types of headaches is linked to cervical structural mechanisms [15–17].

However, previous studies have not yet provided conclusive evidence regarding the association between single or combined cervical MRI findings and headaches [18–20], and the importance of imaging studies investigating headache severity rather than presence has been suggested [21].

In general, MRI is often used to examine potential causes of spinal-related pain and disabilities. However, both the interpretation of MRI findings by health professionals and communication of their clinical relevance must be prioritised to avoid potential negative consequences for patients, such as under- or overtreatment [22]. Although cervical MRI is not routinely indicated for patients presenting primarily with headaches, it is often used in cases of persistent or treatment-resistant symptoms where a cervical aetiology is suspected, and further diagnostic classification is needed. This context provides the opportunity to explore the relationship between the independent variable, cervical MRI findings and the dependent variable, patient-reported headache severity.

This cross-sectional study aims to examine how cervical MRI findings relate to varying severities of headaches in patients with neck pain by (1) investigating the associations between single cervical MRI findings and headache severity and (2) investigating the association between an aggregated score of cervical MRI findings and headache severity.

## Methods

### Study design

This cross-sectional study was based on data from the clinical registry SpineData [23]. The reporting adheres to the Guidelines for Strengthening the Reporting of Observational Studies in Epidemiology (STROBE) [24].

### Study population and settings

The study population in this secondary analysis comprised 611 patients with persistent neck pain who sought multidisciplinary evaluation at a public medical spine clinic, the Spine Centre of Southern Denmark. The referral criteria set included requirements for 6–8 weeks of primary care conservative treatment, such as training, manual therapy, or active relief in parallel with pain medication prior to the referral. Unresponsive to such care or if symptoms worsened, patients were referred to the spine clinic. However, we acknowledge that we do not have data to verify whether this standard was consistently followed in clinical practice for all patients included in the study. Details of the study population have previously been described [25]. In short, patients were included in the study at the entrance to the clinic within the period between January 1st 2011, and December 31st 2014, if they were 18 years or older and had undergone MR imaging of the cervical spine as part of the routine clinical evaluation. The MRI was performed at one of two radiology departments at Lillebaelt Hospital, both of which used identical MRI scanners and standardised interpretation protocols for consistency. MRI assessments were conducted in relation to the current episode of neck pain and typically took place shortly after referral to the spine clinic, generally within approximately one month. Patients were excluded if one of the following pathoanatomies were present: recent acute vertebral fractures, surgical fusions, spinal infections, tumors, or inflammatory spondyloarthropathies. For this study, the incompletion of three or more of the ten items in the Neck Disability Index questionnaire (NDI-10) [26, 27] (included in SpineData and described below) led to exclusion. Also, the NDI-10 item describing headaches should be completed. For those who reported headaches, the characteristics (e.g. duration, location, quality) were unknown, as the primary reason for their referral was neck pain.

### Data sources and collection

#### SpineData

Demographic and patient-reported data were extracted from the clinical, standardised registry SpineData. The patient-reported outcomes (PRO) registry SpineData collects data on symptoms, overall health, and daily functioning. All patients referred to the spine clinic are routinely invited to complete the electronic questionnaires via written correspondence included in their clinical appointment invitation, and asked to do so within one week prior to the clinical entry point [23]. All data is stored in the SpineData registry.

#### MRI

The MRI systems used were Philips 1.0 Tesla, 1.5 Tesla, or 0.2 Tesla. The standard imaging protocols included a sagittal T1-weighted turbo spin-echo, a sagittal short-tau inversion recovery, a sagittal volume isotropic turbo spin echo acquisition, an axial T2-weighted turbo spin-echo, and a reconstructed semi-coronal series from the volume isotropic turbo spin echo acquisition sequence. A MSK-assigned radiologist routinely completed narrative reports for clinical purposes over levels C2-Th1 using EasyViz (Picture Archiving and Communication System, Ontario, Canada), and all reports were double-signed by another MSK radiologist.

The radiology departments followed a consensus document based on international classifications describing the content of a radiologist’s report on an MRI recording of the spine, including the most common MRI findings such as disc degeneration, disc herniation, and central and for aminal stenosis.

### Sample size

The sample size for this study was influenced by convenience, and the study utilised all eligible participants from the original cohort who met our inclusion/exclusion criteria, representing the maximum feasible sample size. Due to the restrictions in accessing the original study population of 1527 participants, the current research is confined to a previously identified study population, as detailed in Jensen et al. [25].

As earlier described, the association of interest was between degenerative cervical MRI findings as independent variables and headache severity as outcome. Precision of the odds ratio estimates was assessed by examining the relative width of the 95% confidence intervals (calculated as the CI width divided by the point estimate). This approach provided a measure of estimate stability relative to the magnitude of the association [28, 29].

### Variables of interest

#### Demographic and clinical variables

The following patient-reported variables collected from the SpineData registry described the study population at a single point of time: age, gender, average intensity of neck and arm pain within the last 14 days (Numeric Rating Scale (NRS) [30, 31]), and health-related quality of life (EuroQoL-5D-3L (EQ-VAS) [32–34]). Furthermore, data on the employment status, as listed in Table 1, and sick leave due to neck or arm pain within the last three months (yes or no) were collected from SpineData. Likewise, information on patient-reported functional neck disability (NDI-10) was collected from SpineData, and the six response options from the headache item (NDI item 5) were reduced to three headache severity (HS) categories. The reduction of responses options was primarily motivated by a desire to enhance the clinical utility of the outcomes. For healthcare professionals, distinguishing between the three severity categories is expected to be significantly more straightforward than navigating the complexity of the six responses options. This simplification is further reinforced by our expectation that the clinical management strategies for headaches will be largely consistent within each of the three categories. As a result, we believe that this reduction will have minimal negative impact on clinical practice. The response options (1) *I have no headaches at all* and (2) *I have slight headaches that come infrequently* were categorised as HS1, “mild headache”, responses (3) *I have moderate headaches that come infrequently,* and (4) *I have moderate headaches that come frequently* were categorised as HS2, “moderate headache”, and (5) *I have severe headaches that come frequently* and 6) *I have headaches almost all the time* were categorised as HS3, “severe headache”.

**Table 1:** Clinical and patient-reported demographic characteristics of the study population (n = 574) stratified by headache severity 1–3 (HS1–HS3)

	Headache severity (HS)		
	HS1 n = 177 (31%)	HS2 n = 249 (43%)	HS3 n = 148 (26%)
**Age (mean (SD)) Range 18–86 (n = 574)**	56.1 (12.2)	51.0 (12.8)	46.9 (12.8)
**Female (n (%)) vs. male (*n* = 574)**	85 (48)	166 (66.7)	109 (73.6)
Typical neck pain intensity (NRS) within the last 14 days (mean (SD)) (n = 573)*	4.3 (2.7)	5.5 (2.3)	6.8 (2.0)
Typical arm pain intensity (NRS) within the last 14 days (median (IQR)/mean (SD)) (n = 570)*	5.0 (5) /4.5 (3.2)	5.0 (5)/4.4 (3.0)	5.0 (5)/4.9 (3.1)
Present work situation (n (%)) (n = 549)*			
Ordinary work: full-time or part-time	87 (52.1)	119 (49.8)	63 (44.1)
Subsidised employment due to reduced work capacity	5 (3.0)	14 (5.8)	10 (7.0)
Studying or undertaking vocational rehabilitation	7 (4.0)	10 (4.1)	7 (4.7)
Unemployed	10 (6.0)	21 (8.8)	10 (7.0)
Receiving a disability pension#	8 (4.8)	16 (6.7)	18 (12.6)
**Receiving a retirement pension**	44 (26.3)	37 (15.5)	15 (10.5)
**Not in employment (homemaker/other reason)**	6 (3.6)	22 (9.2)	20 (14.0)
Sick-leave for neck pain or arm pain within the last 3 months (n (%)) vs. no sick-leave (n = 398)*	40 (35)	73 (41)	46 (44)
Self-reported health (EuroQoL-5D-3L (EQ-VAS)) (mean (SD)) (n = 562)*	58.2 (24.8)	53.3 (23.3)	45.0 (23.2)
**Neck Disability Index score (mean (SD)) (n = 574)**	27.8 (16.6)	38.3 (13.4)	50.0 (14.3)

*Percentages are calculated based on non-missing data. Sample sizes vary by variable due to single missing values

Valid sample sizes per group for each variable are as follows:

Work situation: HS1 = 167, HS2 = 239, HS3 = 143 (total = 549)

Sick leave: HS1 = 114, HS2 = 179, HS3 = 105 (total = 398)

Please refer to valid n’s for accurate interpretation of percentages

^#^Individuals over the age of 40 and having permanently reduced capacity to work in a substantial degree unabling regular work or subsidised employment

HS: Headache severity; SD, standard deviation; NRS, Numeric Rating Scale (0–10); IQR, interquartile range; EQ-5D, Euro-QoL-5D

HS1: “I have no headaches, or I have slight headaches, which come infrequently”; HS2: “I have moderate headaches, which come infrequently or frequently”; HS3: “I have severe headaches, which come frequently, or I have headaches almost all the time”

Bold indicates Bonferroni corrected *p*-value < 0.006

#### MRI variables

Data on the MRI variables were based on the coding of the MRI narrative reports. Data extraction and quantification of the MRI data was based on the work of Kent et al. [35]and has previously been described by Jensen et al. [25]; For the purpose of the original work, a set of coding rules was developed to record the presence or absence of an MRI finding on each of the cervical segmental levels from C2-Th1, and the reviewers of the narrative reports (and thus coders) were six second-year chiropractic master students who had finished their imaging course of 16 ECTS points (European Credit Transfer and Accumulation System). All coding was blinded, consensus was required, and reliability studies were conducted to ensure the data quality [25]. The MRI findings of interest were disc degeneration, disc protrusion, disc herniation, nerve root compromise, facet joint arthrosis, uncovertebral arthrosis, foraminal stenosis, central stenosis, and vertebral endplate changes (VESC) type 1 and 2. The nomenclature used to evaluate the MRI findings from the narrative reports is described in detail in Appendix 1. The MRI findings describe degenerative changes affecting the discovertebral complex, the central spinal canal, and the neural foraminal spaces, which are commonly described in studies of the cervical spine [36–38]. Further, the findings are often referred to in routine everyday practice which support comparability with previous studies and enhances the clinical relevance of our results.

The MRI findings reflect the state of degeneration at a single point in time.

### Statistical analysis

Normally distributed demographic and clinical variables were described as means, non-parametric data as medians, and categorical data were expressed as proportions. Associations between study population characteristics and the HS were appropriately estimated using one-way ANOVA, Kruskal–Wallis, or Chi-square tests. Characteristics for patients included and those excluded were compared to identify potential selection bias. Post hoc Bonferroni adjustments were included in the descriptive analyses to avoid Type 1 errors.

Accounting for age as a confounder, as age is associated with both the presence of degenerative MRI findings and headache [2, 39, 40], crude and age-adjusted univariate ordinal logistic regression analyses estimated the associations between the presence of single MRI findings and the HS. The assumptions of proportional odds were adequately tested, and the MRI variables were examined for intercorrelation, and variables with moderate or strong correlation (r > 0.5) were excluded from the following analyses. All remaining MRI variables were included in the multivariable backward stepwise ordinal logistic age-adjusted regression analysis. The Likelihood ratio test and Akaike Information Criterion (AIC) were used for assessing the stepwise selection. The stepwise regression analysis was, however, terminated if or when the remaining variables had odds ratios (ORs) with potential clinical significance. An estimated association with an OR or hazard ratio (HR) greater than 1.5 has been used in a previous study as an indication of potential clinical significance [41]. In our study, we re-used this arbitrary threshold (OR > 1.5) and its reciprocal (OR < 0.7) as criteria for selecting MRI variables for inclusion in the overall score. The confidence intervals were tested for multiplicity using bootstrapping (1000 reps).

An aggregated score was generated on single MRI findings summed across all levels. Each type of MRI finding was counted as one finding only, regardless of its frequency (e.g., disc herniations at three spinal levels and nerve root compromise at two levels made an aggregated score of two). MRI findings were only included in the aggregate score if they were associated with the HS in a potentially clinically important manner in the multivariable regression analysis. A sensitivity analysis was conducted to assess the robustness of the OR estimates for the aggregate score. OR estimates based on a score including all MRI variables were compared to estimates based on a score derived from the multivariable model.

Estimates for the association between the aggregated score and the HS were established using univariate regression analysis. All associations were expressed as odds ratios (ORs) with 95% confidence intervals (CIs) calculated using the exact method (Clopper–Pearson interval).

The analyses were performed using Stata 17.0 (StataCorp, College Station, Texas).

## Results

### Study population

**Fig. 1:**
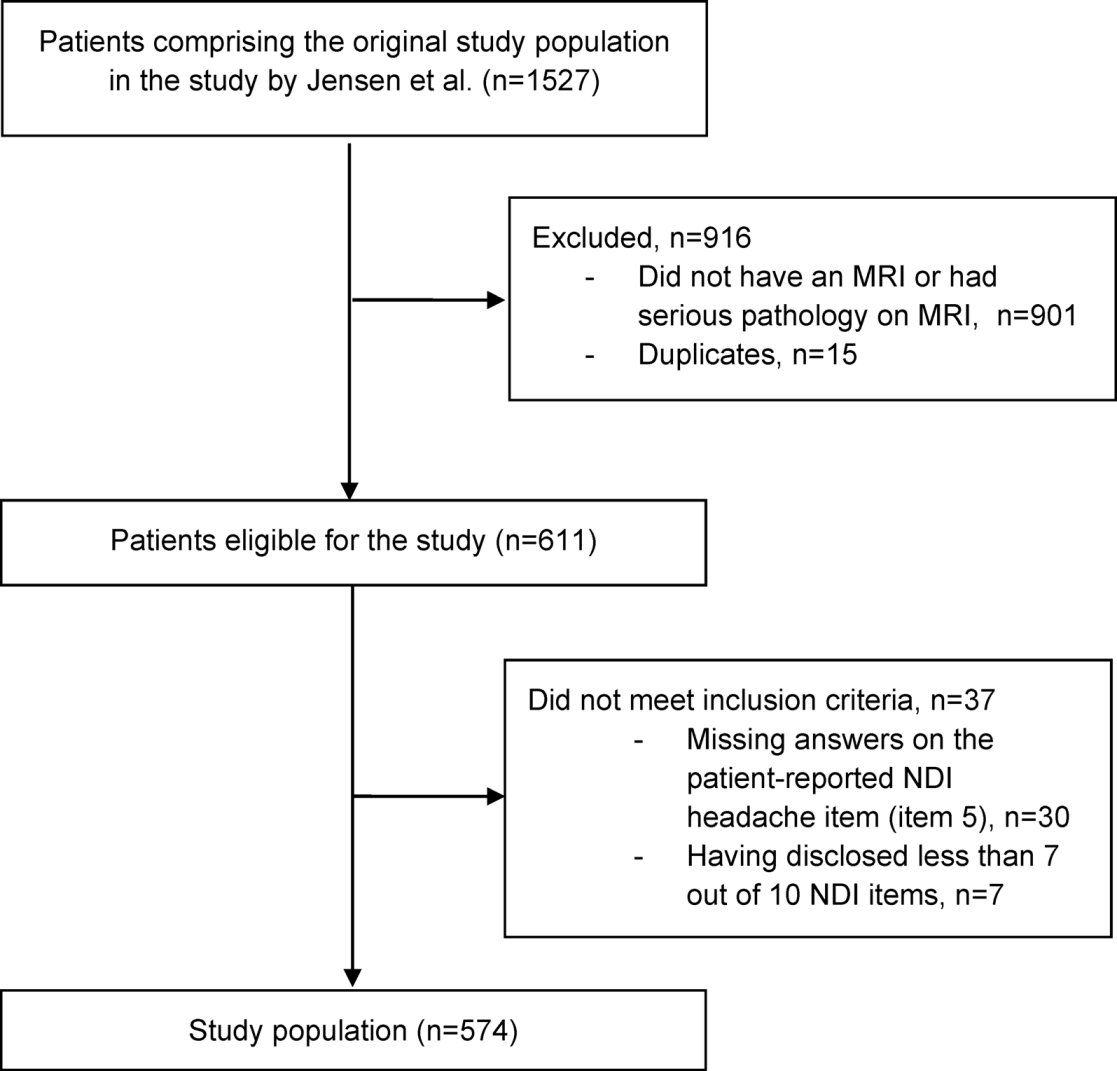
Flowchart describing the study population selection process

Of the 611 eligible participants identified from the original study population, 37 (6%) were excluded due to either missing answers on the NDI headache item (item 5) (n = 30) or having disclosed less than 7 out of 10 NDI-10 items (n = 7), comprising a total study population of 574 participants. (Fig. 1: Flowchart describing the study population selection process). A comparison of characteristics between the individuals included and those excluded showed limited differences (Appendix 2). The excluded individuals (n = 37) were statistically significantly less likely to attend full-time or part-time work compared to those included.

Appendix 3 presents the clinical and patient-reported demographic characteristics of the study population stratified by responses 0 to 5 on item 5 of the NDI-10.

The distribution of responses among the six categories (NDI-10, item 5) were as follows; I have no headaches at all n = 91 (16%), I have slight headaches that come infrequently n = 86 (15%), I have moderate headaches that come infrequently n = 75 (13%), I have moderate headaches that come frequently n = 174 (30%), I have severe headaches that come frequently n = 65 (11%), and I have headaches almost all the time n = 86 (14.5%).

### Demographic and clinical characteristics

The demographic and clinical characteristics of the study population, including data completeness stratified by headache severity, are presented in Table 1. The proportion of participants in HS1, HS2, and HS3 were 31%, 43%, and 26%. Higher headache severity (HS) was associated with several demographic and clinical factors. Females had increased odds of higher HS compared to males, with ORs of 1.8 (95% CI 1.3–2.5) for HS2 versus HS1 and 2.3 (95% CI 1.6–3.4) for HS3 versus HS1. Younger age was linked to higher HS, with each 10-year decrease associated with ORs of 1.5 (95% CI 1.2–1.9) for HS2 vs HS1 and 1.7 (95% CI 1.3–2.2) for HS3 vs HS1. Lower quality of life (EQ-5D) scores were associated with higher HS: compared to HS1, HS3 had an OR of 2.0 (95% CI 1.4–2.9), and HS2 had an OR of 1.4 (95% CI 1.1–1.8). Higher neck disability (NDI) scores and neck pain intensity were also positively associated with higher HS (ORs ranging from 1.6 to 2.1). Participants classified as homemakers or others had 1.9 times higher odds (95% CI 1.3–2.8) of being in HS3 versus HS1, while those receiving retirement pensions had reduced odds (OR 0.5, 95% CI 0.3–0.8) of higher HS.

### Associations between single cervical MRI findings and headache severity

Table 2 lists the number of MRI findings across the assessed levels (C2-Th1). The most common findings were foraminal stenosis (present in n = 439 participants (76.5%)), disc degeneration (n = 388 (67.6%)), and disc protrusion (n = 290 (50.5%)). The least common were VESC type 2 (n = 34 (6%)) and nerve root compromise (n = 14 (2%)).

**Table 2:** Univariate and multivariable analysis of the associations between single cervical Magnetic Resonance Imaging (MRI) findings and the outcome variable patient-reported headache severity. Associations are presented as Odds Ratios (OR) with 95% confidence intervals (CI). The ORs indicate the odds of having a higher headache severity category in the presence versus absence of each MRI finding

High headache severity (HS2-3) vs. low (HS1)	Number of MRI findings	% (of total n = 574)	Univariate analysis		Multivariable analysis	
					Three HS categories	Six HS categories
			OR (95% CI)		OR (95% CI)	OR (95% CI)
			Crude	Age-adjusted	Age-adjusted	
Participants with single cervical MRI findings						
VESC type 1	143	24.9	0.79 (0.56–1.13)	1.03 (0.71–1.48)	Excluded	Excluded
VESC type 2	34	5.9	**0.333 (0.17–0.66)**	**0.42 (0.21–0.84)**	**0.44 (0.22–0.88)**	**0.45 (0.23–0.85)**
Disc degeneration	388	67.6	**0.59 (0.43–0.82)**	0.86 (0.61–1.22)	Excluded	Excluded
Disc herniation	90	15.7	0.66 (0.43–1.00)	**0.64 (0.42–0.98)**	0.67 (0.43–1.03)	0.70 (0.47–1.04)
Disc protrusion	290	50.5	0.78 (0.58–1.06)	0.95 (0.69–1.29)	Excluded	Excluded
Nerve root compromise	14	2.4	1.14 (0.40–3.21)	1.05 (0.37–2.99)	Excluded	Excluded
Facet joint arthrosis	211	36.8	**0.61 (0.45–0.84)**	0.99 (0.69–1.41)	Excluded	Excluded
Uncovertebral arthrosis	424	73.9	**0.46 (0.32–0.65)**	0.75 (0.50–1.12)	Excluded	Excluded
Central stenosis	215	37.5	**0.59 (0.43–0.81)**	0.79 (0.57–1.10)	Excluded	Excluded
Foraminal stenosis	439	76.5	**0.372 (0.26–0.54)**	**0.62 (0.40–0.95)**	0.68 (0.44–1.06)	0.71 (0.47–1.07)

Bold indicates *p*-value < 0.05

OR, Odds Ratio; CI, Confidence Interval; VESC, Vertebral Endplate Signal Changes

The univariate analysis showed that, except for nerve root compromise (OR 1.14 (95% CI 0.40–3.21)), all crude ORs were below one (Table 2), indicating a decrease between 21 and 67% in the odds of having moderate or severe headache if the finding was present compared to if the finding was absent. After adjusting for age, VESC type 2 (OR 0.42 (95% CI 0.21–0.84)) and foraminal stenosis (OR 0.62 (95% CI 0.40–0.95)) remained significantly associated with headache severity.

Due to intercorrelation between MRI variables, uncovertebral and facet joint arthrosis were excluded prior to the multivariable analysis. The MRI findings with the strongest age-adjusted association with the HS were VESC type 2, disc herniation, and foraminal stenosis (Table 2). The three variables informed the aggregate score. All three had OR > 1.50 or < 0.70; however, the associations for disc herniation and foraminal stenosis were not statistically significant. While the OR suggested stronger associations for VESC type 2, the low prevalence (n = 34) led to wide confidence intervals and therefore limited precision. This introduces uncertainty in the estimate, and the apparent statistical significance might be overestimated due to the low prevalence. We therefore advise caution when interpreting this finding. Bootstrapping did not indicate multiplicity (data not shown).

We also repeated the multivariable regression analyses using the NDI item 5 with six categories as the dependent variable, allowing us to examine whether information was lost with the reduction of categories through comparison of OR’s (Table 2). The estimates of the OR’s were broadly consistent with those produced using the three categories.

### Associations between the aggregated score of cervical MRI findings and the headache severity

The aggregated score was informed by VESC type 2, disc herniation, and foraminal stenosis (range 0–3). 21% of participants (n = 123) had no cervical MRI findings, 60% (n = 344) had one finding, 18% (n = 102) had two, and 1% (n = 5) had all three types of MRI findings. Because of the low prevalence of the latter, the groups with two and three findings were pooled.

Participants with more present cervical MRI findings had significantly decreased odds of higher HS than participants with fewer or no MRI findings (Table 3). Having 2–3 different types of MRI findings or one type of finding was associated with 2.5 and 1.7 times less risk of higher HS compared to having no MRI findings (OR 0.40 (95% CI 0.23–0.68) and OR 0.56 (95% CI 0.35–0.88), respectively).

**Table 3:** Results of univariate analysis of the associations between the aggregate score and the patient-reported headache severity. Associations presented as Odds Ratios (OR) with 95% confidence intervals (CI)

	Univariate analysis	
	OR (95% CI)	
	Crude	Age-adjusted
Aggregate score†		
0 findings (n = 123 (21%))	Reference category	Reference category
1 finding present (n = 344 (60%))	**0.339 (0.23–0.50)**	**0.56 (0.35–0.88)**
2–3 findings present (n = 107 (19%))	**0.258 (0.16–0.42)**	**0.40 (0.23–0.68)**

^†^The aggregated score was informed by the cervical MRI findings Vertebral Endplate Signal Changes (VESC) type 2, disc herniation, and foraminal stenosis

OR, Odds Ratio; CI, Confidence Interval. Bold indicates *p*-value < 0.05

Testing the robustness of the OR estimates derived from the aggregate score by including all MRI available variables (range zero to seven) resulted in similar magnitudes and directions of all OR estimates (data not shown). These findings supported our decision to pool the groups with two and three MRI findings.

The post hoc precision assessments of the sample size found that the majority of odds ratios exhibited moderate confidence interval widths (0.5–0.8), reflecting reasonable precision given the fixed sample size and prevalence of findings (data not shown). The exception was nerve root compromise, which had a wide confidence interval (2.62) likely due to its low prevalence, indicating limited precision. The aggregated MRI score demonstrated relatively narrower confidence intervals (1 finding present: 0.53 and 2–3 findings present: 0.45).

## Discussion

The results of the study indicated that having one or more cervical MRI findings was associated with less severe headaches compared to having no findings.

The aggregate score constructed in this study was based on the cervical MRI findings assessed to be of potential clinical relevance after a multivariable stepwise regression analysis. We recognize that aggregate scores can be constructed differently, which is a methodological consideration, and that the choice of construction may affect the magnitude of the associations. In our study, however, this does not seem to be the case, as the associations were unchanged in the sensitivity analysis regarding both magnitude and direction.

Using different designs, other studies [41–44] have similarly investigated the clinical importance of cumulative scores of spinal MRI findings compared to single findings and found associations of varying magnitudes and orientations. However, these studies were using back pain as the outcome. To the best of our knowledge, this is the only study that has examined the severity of headaches as the outcome, and a direct comparison of the present findings is, therefore, not possible.

Nevertheless, we expected positive associations between cervical MRI findings and symptoms corresponding to those demonstrated by Hancock et al. [41] due to similar anatomy. Instead, negative associations were found between the presence of both single and aggregated scores of cervical MRI findings and the headache severity. This was a counterintuitive finding, as degenerative changes within the spine are associated with local pain to some extent [45–47], and we expected local neck pain to cause secondary headaches. These inverse associations, with odds ratios ranging from approximately 0.3 to 0.7, suggest a reduction in the likelihood of more severe headaches when such MRI findings were present. While not all associations reached statistical significance, the consistent negative direction across multiple findings strengthens the internal coherence of the results.

Given that participants were referred primarily for persistent neck pain, there is a possibility of reporting bias, with patients focusing more on neck symptoms than on possible co-existing headaches. This may have attenuated any positive associations that might otherwise have been detected.

More importantly, compared to the crude analysis, the observation that the overall magnitude of ORs decreased after age adjustment suggests that age explains a central part of our somewhat unexpected findings. Older individuals in the study were more likely to exhibit degenerative changes on MRI but reported less severe headaches. This aligns with epidemiological evidence that the headache prevalence decreases with age [48].

Other known, but not accounted for, biological factors affecting the prevalence and possibly severity of headaches include hormone levels and gender [2, 48]. Moreover, psychological factors such as anxiety, stress and depression are more frequent among patients with headaches and they are identified as common headache triggers [48], and more social factors such as socioeconomics and educational level have been associated with the prevalence of headache [49]. These unmeasured factors may have affected both the reporting of headache severity and its actual experience, potentially contributing to the observed inverse associations. For instance, individuals with greater psychosocial stress might report more severe headaches regardless of MRI findings, thereby influenced the magnitude and direction of the associations. However, the aim of this study was to explore the association between MRI findings only and HS, as this has not previously been done.

### Methodological considerations

Internal validity is influenced by measurement limitations. The MRIs did not include the upper cervical spine (C0–C1 and C1–C2), which may be more directly implicated in some headache types.

When cervical MRIs are conducted in a clinical setting because of spinal pain, the narrative descriptions conducted by radiologists do not routinely include the C0-C1 and C1-C2 levels, as the main subjects of the examination are the disc complex. The aim of the present study was to examine the association between patient-reported headache and cervical MRI findings within a population of patients with neck pain, and the narrative reports were subsequently limited to only include C2-Th1, which would be the case in similar clinical populations. The proposed pathophysiology of, for example, the cervicogenic headache is a complex interaction between the spinal nerves C1–C3 and the trigeminal afferents [50, 51], and pericranial tenderness has been considered important in the pathophysiology of tension-type headaches [5]. An unpublished systematic literature review has, however, identified only a small number of existing studies on upper cervical MRI findings and headache (n = 3), all of which had small sample sizes and uncertainties in the results [52]. In this study, the findings most often described in the upper spine would likely qualify for exclusion due to the pathological aetiology: Transverse ligament rupture, atlantoaxial instability, fractures, inflammatory or infectious conditions, and tumors. We believe that, in future studies, including the upper cervical levels, C0-C1 and C1-C2, could enhance our understanding and contribute valuable insights to the field.

The study was limited by including only one clinical setting and a population of patients with persistent neck pain as the primary complaint. As the headache type, location, duration, and quality were unknown, this study describes overall headache disorders only. While some headaches present secondary to severe and life-threatening conditions [53], the headaches reported in this study were perceived to be largely non-acute based on the referral criteria set by the spine clinic, as mentioned in the Methods section. However, we cannot rule out severe illnesses causing headaches among the included patients. These factors may limit the generalizability and precision of the results and should, therefore, be interpreted cautiously. In future studies, it would be relevant to test the associations in a population with headache as the primary complaint.

As noted in the Methods section, the original cohort described by Jensen et al. [25] included 1,527 participants, but due to data access limitations, we were unable to retrieve the full dataset from the original study, which may affect the generalizability of our findings.

To our knowledge, this is the first study to examine the association between cervical MRI findings and patient-reported headache severity, expanding the scope beyond prior investigations of associations between MRI findings and intensity of back pain. The findings of this study do not significantly enhance our understanding of the underlying causes of headaches. However, they offer insights into the association between commonly observed degenerative changes in the cervical spine and headaches, and likely reflect true patterns within the studied clinical population. The findings of this study suggest a potential association between present cervical MRI findings and less severe headaches among patients with persistent and dominant neck pain referred to secondary care. These results should be interpreted within the context of the selected population. Future studies are needed to assess whether similar associations are observed in populations with different symptom profiles, including those with dominant headache symptoms.

## Conclusions

This study showed that, among patients with persistent neck pain, the presence of degenerative MRI findings in the cervical spine was associated with lower headache severity. Moreover, the association between an aggregated score of MRI findings and headache severity was stronger than that of single findings. These results reflect associations observed within a selected clinical population and highlight the need for further research in populations with differing symptom profiles.

## Supplementary Information

Below is the link to the electronic supplementary material.

Supplementary Material 1

Supplementary Material 2

Supplementary Material 3

## Data Availability

No datasets were generated or analysed during the current study.

## Abbreviations

MRI: Magnetic resonance imaging
HS: Headache severity
NRS: Numeric rating scale
EQ: Euro QoL (Quality of Life)-5D
VAS: Visual analogue scale
VESC: Vertebral endplate changes
NDI-10: Neck disability index questionnaire
OR: Odds ratio
CI: Confidence Interval
