# Supplementary material for: Association between cervical MRI findings and patient-reported severity of headache in patients with persistent neck pain: a cross-sectional study
Source: Chiropr Man Therap. 2025 Sep 1;33:38. doi: 10.1186/s12998-025-00600-4 (PMC12403482; doi:10.1186/s12998-025-00600-4)
Supplement: Supplementary file 1 — Supplementary Material 1 [file 12998_2025_600_MOESM1_ESM.docx]

Appendix 1: Magnetic Resonance Imaging (MRI) variables of interest and evaluation nomenclature

| Variables | Evaluation nomenclature |
| --- | --- |
| Disc degeneration (1) | None  Mild = Slightly reduced disc height and/or reduced signal of the nucleus.  Moderate = Reduced disc height and reduced signal of the nucleus.  Severe = Collapsed disc space. |
| Disc herniation (2) | None  Broad-based herniation: 25-50% = 90-180 degrees  Focal herniation: <25% = < 90 degrees. Width>Depth  Extrusion: Depth>Width or ascending/descending behind corpora  Sequestration: No connection between tissue and the disc to which it belongs |
| Disc protrusion (2) | None Present: >50%, including focal herniations and extrusions |
| Nerve root compromise (3) | None  Touch  Displaced  Compressed |
| VESC Type 1 and 2 (4) | None  Present: Above upper and lower endplate level. Not including corner lesions.  VESC Type 1: hypointense T1 signal, hyperintense T2 & STIR signal  VESC Type 2: hypointense STIR signal, hyperintense T1 & T2 signal |
| Facet joint arthrosis (5, 6) | None  Present: 1) joint space narrowing and/or 2) osteophytes. |
| Uncovertebral arthrosis (5, 6) | None  Present: 1) joint space narrowing and/or 2) osteophytes. |
| Foraminal stenosis (7) | None  Mild (relative) = Less space than normal, but visible perineural fat signal around the nerve root. < 50% area reduction. Mild stenosis is described as a normal condition. Moderate (relative) = Less space than normal, but visible perineural fat signal around the nerve root. > 50% area reduction.  Severe (absolute) = Reduction of foramen area and no visible perineural fat. |
| Central stenosis (8) | None Mild (relative) = Less space than normal, but visible fluid signal between nerve roots. < 50% area reduction. Mild stenosis is described as a normal condition.  Moderate (relative) = Less space than normal, but visible fluid signal between nerve roots. > 50% area reduction.  Severe (absolute): No visible fluid signal around nerve roots or medulla spinalis |
| VESC = Vertebral Endplate Signal Changes | |

References

1. Pfirrmann CW, Metzdorf A, Zanetti M, Hodler J, Boos N. Magnetic resonance classification of lumbar intervertebral disc degeneration. Spine. 2001;26(17):1873-8.

2. Fardon DF, Milette PC. Nomenclature and classification of lumbar disc pathology. Recommendations of the Combined task Forces of the North American Spine Society, American Society of Spine Radiology, and American Society of Neuroradiology. Spine. 2001;26(5):E93-e113.

3. Pfirrmann CW, Dora C, Schmid MR, Zanetti M, Hodler J, Boos N. MR image-based grading of lumbar nerve root compromise due to disk herniation: reliability study with surgical correlation. Radiology. 2004;230(2):583-8.

4. Modic MT, Steinberg PM, Ross JS, Masaryk TJ, Carter JR. Degenerative disk disease: assessment of changes in vertebral body marrow with MR imaging. Radiology. 1988;166(1 Pt 1):193-9.

5. Weishaupt D, Zanetti M, Boos N, Hodler J. MR imaging and CT in osteoarthritis of the lumbar facet joints. Skeletal radiology. 1999;28(4):215-9.

6. Pathria M, Sartoris DJ, Resnick D. Osteoarthritis of the facet joints: accuracy of oblique radiographic assessment. Radiology. 1987;164(1):227-30.

7. Park HJ, Kim SS, Chung EC, Lee SY, Park NH, Rho MH, et al. Clinical correlation of a new practical MRI method for assessing cervical spinal canal compression. AJR Am J Roentgenol. 2012;199(2):W197-201.

8. Kang Y, Lee JW, Koh YH, Hur S, Kim SJ, Chai JW, et al. New MRI grading system for the cervical canal stenosis. AJR Am J Roentgenol. 2011;197(1):W134-40.
