# Supplementary material for: Association between cervical MRI findings and patient-reported severity of headache in patients with persistent neck pain: a cross-sectional study
Source: Chiropr Man Therap. 2025 Sep 1;33:38. doi: 10.1186/s12998-025-00600-4 (PMC12403482; doi:10.1186/s12998-025-00600-4)
Supplement: Supplementary file 2 — Supplementary Material 2 [file 12998_2025_600_MOESM2_ESM.docx]

Appendix 2. Clinical and patient-reported demographic characteristics for patients included and those excluded, in total n=611.

|  | **Included**  n = 574 (94%) | **Excluded**  n = 37 (6%) | ***P*** |
| --- | --- | --- | --- |
| Age (mean (SD)) (range 18-86) (n=611) | 51.4 (13.1) | 56.8 (14.2) | 0.02 |
| Female vs. male (n (%)) (n=611) | 360 (63) | 23 (62) | 0.54 |
| Typical neck pain intensity within the last 14 days, NRS (mean (SD)) (n=590)* | 5.5 (2.5) | 5.8 (3.0) | 0.60 |
| Typical arm pain intensity within the last 14 days, NRS (mean (SD) (n=587)* | 4.5 (3.1) | 2.7 (3.2) | 0.02 |
| Present work situation (n (%)) (n=562)*  **Ordinary job (full-time or part-time)**  Subsidised employment due to reduced work capacity  Studying or undertaking rehabilitation  Unemployed  Receiving disability pension#  Receiving retirement pension  Housemaker, or other | 269 (49.0)  29 (5.3)  24 (4.4)  41 (7.5)  42 (7.7)  96 (17.5)  48 (8.7) | 4 (30.8)  1 (7.7)  0  4 (30.8)  1 (7.7)  3 (23.1)  0 | <0.01  0.45  0.22  0.29  0.25  0.12  0.04 |
| Sick-leave for neck pain or arm pain within the last 3 months vs. no sick-leave (n (%)) (n=407)* | 158 (39.7) | 3 (33.3) | 0.49 |
| Self-reported health (EQ-5D-3L thermometer) (mean (SD)) (n=577)* | 52.6 (24.2) | 59.2 (32.0) | 0.31 |
| Neck Disability Index score (mean (SD)) (n=550)* | 38.1 (16.9) | 21.4 (11.3) | 0.02 |

Note: Percentages are calculated based on non-missing data. Sample sizes vary by variable due to single missing values.
Valid sample sizes per group for each variable are as follows:
Work situation: Included = 549, Excluded = 13 (total = 562)

Sick leave: Included = 398, Excluded = 9 (total = 407)

Please refer to valid n’s for accurate interpretation of percentages.

# Individuals over the age of 40 and having permanently reduced capacity to work in a substantial degree unabling regular work or subsidised employment.

SD, standard deviation; NRS, Numeric Rating Scale (0-10); EQ-5D, Euro-QoL-5D.
Bold indicates Bonferroni corrected p-value < 0.006 
