# Supplementary material for: Association between cervical MRI findings and patient-reported severity of headache in patients with persistent neck pain: a cross-sectional study
Source: Chiropr Man Therap. 2025 Sep 1;33:38. doi: 10.1186/s12998-025-00600-4 (PMC12403482; doi:10.1186/s12998-025-00600-4)
Supplement: Supplementary file 3 — Supplementary Material 3 [file 12998_2025_600_MOESM3_ESM.docx]

Appendix 3: Clinical and patient-reported demographic characteristics of the study population (n=574) stratified by Neck Disability Index (NDI-10), item 5 response options 0-5.

|  | **Neck Disability Index (NDI-10), item 5** | | | | | |
| --- | --- | --- | --- | --- | --- | --- |
|  | **0**  n = 91 (16%) | **1**  n = 86 (15%) | **2**  n = 75 (13%) | **3**  n = 174 (30%) | **4**  n = 65 (11%) | **5**  n = 83 (15%) |
| Age (mean (SD))  Range 18-86 (n=574) | 57 (12.1)) | 55 (12.4) | 51.0 (13.8) | 51 (12.3) | 48 (11.4) | 46 (13.8) |
| Female (n (%)) vs. male (n=574) | 41 (45) | 44 (51) | 50 (67) | 116 (67) | 44 (68) | 65 (78) |
| Typical neck pain intensity (NRS) within the last 14 days (mean (SD)) (n=573)* | 3.6 (2.6) | 5.1 (2.7) | 5.5 (2.4) | 5.6 (2.3) | 6.8 (2.0) | 6.8 (2.0) |
| Typical arm pain intensity (NRS) within the last 14 days (median (IQR)/mean (SD)) (n=570)* | 5.0 (5)/4.5 (3.2) | 5.0 (5)/4.6 (3.1) | 4.0 (6.0)/4.2 (3.1) | 5.0 (5)/4.5 (3.0) | 5.0 (5)/ 4.8 (3.0) | 5.0 (5)/4.9 (3.3) |
| Present work situation (n (%))  (n=549)*  Ordinary work: full time or part-time  Subsidised employment due to reduced work capacity  Studying or undertaking vocational rehabilitation  Unemployed  Receiving a disability pension#  Receiving a retirement pension  Not in employment (homemaker/other reason) | 41 (47.7)  1 (1.2.)  4 (4.4)  8 (9.3)  3 (3.5)  25 (29.1)  4 (4.4) | 46 (56.8)  4 (4.9)  3 (3.5)  2 (2.5)  5 (6.2)  19 (23.5)  2 (2.3) | 37 (51.4)  4 (5.6)  4 (5.3)  5 (6.9)  2 (2.8)  15 (20.8)  5 (6.7) | 82 (49.1)  10 (6.0)  6 (3.5)  16 (9.6)  14 (8.4)  22 (13.2)  17 (9.8) | 31 (51.7)  3 (5.0)  3 (4.6)   5 (8.3)  6 (10.0)  6 (10.0)  6 (9.2) | 32 (38.6)  7 (8.4)  4 (4.8)  5 (6.0)  12 (14.5)  9 (10.8)  14 (16.9) |
| Sick-leave for neck pain or arm pain within the last 3 months vs. no sick-leave (n (%)) (n=398)* | 17 (30) | 23 (40) | 26 (50)) | 47 (37) | 22 (49) | 23 (38) |
| Self-reported health (EQ-5D-3Lthermometer) (mean (SD)) (n=562)* | 63.1 (23.6) | 53.0 (25.2) | 53.4 (24.3) | 53.3 (23.0) | 48.0 (22.3) | 42.7 (23.7) |
| Neck Disability Index score (mean (SD)) (n=574) | 23.7 (14.5) | 32.2 (17.5) | 34.5 (11.4) | 40.0 (13.9) | 47.1 (12.8) | 52.3 (15.1) |

*Note: Percentages are calculated based on non-missing data. Sample sizes vary by variable due to single missing values.
Valid sample sizes per group for each variable are as follows:
Work situation: 0 = 86, 1 = 81, 2 = 72, 3 = 167, 4 = 60, 5 = 83 (total = 549)

Sick leave: 0 = 57, 1 = 57, 2 = 52, 3 = 127, 4 = 45, 5 = 60 (total = 398)

Please refer to valid n’s for accurate interpretation of percentages.

# Individuals over the age of 40 and having permanently reduced capacity to work in a substantial degree unabling regular work or subsidised employment.

SD, standard deviation; NRS, Numeric Rating Scale (0-10); IQR, interquartile range; EQ-5D, Euro-QoL-5D.
